# Haemolymphatic Parameters in Two Aquaculture Crustacean Species *Cherax destructor* (Clark, 1836) and *Cherax quadricarinatus* (Von Martens, 1868)

**DOI:** 10.3390/ani12050543

**Published:** 2022-02-22

**Authors:** Manuela Mauro, Vincenzo Arizza, Marco Arculeo, Alessandro Attanzio, Paola Pinto, Pietro Chirco, Giampaolo Badalamenti, Luisa Tesoriere, Mirella Vazzana

**Affiliations:** Dipartimento di Scienze e Tecnologie Biologiche, Chimiche e Farmaceutiche (STEBICEF), Università di Palermo, 90128 Palermo, Italy; manuela.mauro01@unipa.it (M.M.); vincenzo.arizza@unipa.it (V.A.); marco.arculeo@unipa.it (M.A.); alessandro.attanzio@unipa.it (A.A.); paola.pinto@unipa.it (P.P.); pietro.chirco@libero.it (P.C.); giampaolobadalamenti@libero.it (G.B.); luisa.tesoriere@unipa.it (L.T.)

**Keywords:** crayfish, *Cherax* spp., total haemocytes count, total protein, aquaculture plant

## Abstract

**Simple Summary:**

The spread of freshwater crustacean farms, in particular of the Australian species Cherax, has been widespread in recent years and has aroused particular interest at an economic level. Knowledge of the basic levels of some biochemical parameters becomes particularly important to understanding the health status of the animals and, therefore, for the maintenance of aquaculture facilities. In this study, the values of some biochemical parameters of two species of Cherax, *Cherax destructor* and *Cherax quadricarinatus*, reared in an Italian aquaculture plant, were evaluated for the first time. These parameters should contribute to assessing the health status of these animals on a farm and to understanding if they will be affected by stressful conditions or not.

**Abstract:**

In the last few years, there has been a notable development in the breeding of freshwater shrimp (astaciculture), which involved various species and in particular, the two Australian *Parastacidae* species, *Cherax destructor* and *Cherax quadricarinatus*. Information about the haemolymphatic parameters of these two species is fragmentary, and filling these gaps becomes important given their importance in aquaculture. Cellular and biochemical parameters were analyzed in both species to create a reference baseline for these parameters to identify the state of welfare or suffering of these animals. The results showed that the total haemocyte count, haemocyte subpopulations, enzymatic activities and pH are similar between the two species, while total protein and osmolality are higher in *C. destructor* than *C. quadricarinatus*. Knowledge of these parameters could assist in evaluating the good health status of these species kept in aquaculture facilities.

## 1. Introduction

It is well known that marine or freshwater vertebrates and invertebrates play an important role as environmental bioindicators [1,2,3,4,5] and as a source of bioactive molecules for the treatment of medical applications [6,7,8,9,10,11,12]. The red shrimp, *Cherax quadricarinatus*, is a native species of northern Australia and southern New Guinea and has become one of the most important crustacean species for aquaculture purposes. It is among the largest freshwater decapods, matures early, and females can lay over a thousand eggs in a single brood and has a broad environmental tolerance [13]. All of these characteristics make it a highly appreciated species for aquaculture all over the world, to the point of becoming the second most economically important crayfish species after *Procambarus clarkii* [13]. In addition to this species, the yabby (*Cherax destructor*), the most widespread freshwater shrimp species in Australia, also showed a very high potential for aquaculture purposes [14,15]. Thanks to these characteristics, a few years ago, the breeding of these two species has spread to Europe and recently to Italy (Sicily). Although some commercial yabby farms have experienced significant disease problems in the past, such as the pathogenicity of Vibrio mimicus [16] or cases of ectosymbiotic Temnocephala flatworms [17], today, the health status of the European yabbies is still not well known. Conversely, both *Cherax* species have been studied from various points of view, such as the effects of dietary supplementation [18,19,20], changes in haemolymphatic parameters following molting [21], effects of pollutants [22,23], and spatial distribution [24]. Despite this, to date, no one has analyzed the basic cellular and biochemical parameters of the haemolymph of these two species kept in optimal breeding conditions. Stress in crustaceans has been evidenced by measurements of biochemical and behavioral (physical injuries) parameters [25]. In fact, physical and chemical aquatic environmental stressors [26,27,28] are responsible for important changes in crustaceans, such as (i) the alteration of the endocrine system, influencing the release of neuropeptide crustacean hyperglycaemic hormone (CHH) and glucose [25]; (ii) the regulation of the ecdysone receptor [29,30,31], which is important for growth, development, reproduction and regeneration; (iii) the total protein levels in haemolymph [32]; and (iv) the total and differential haemocytes’ count [1] and pH values, and osmolality in the haemolymph [33,34]. All these parameters could be useful to control animal welfare and maintain good conditions during breeding. For these reasons, in this study, we report a preliminary analysis of some haemolymphatic parameters of the two Cherax species reared in an aquaculture plant located in the east of Sicily. These parameters could provide information on the proper maintenance and animal welfare of the two freshwater shrimp species and allow for the enhancement of the production of these two species.

## 2. Materials and Methods

### 2.1. Animals

A total of 30 adult individuals for each of the 2 species (*C. quadricarinatus* and *C. destructor*) were used to evaluate the parameters of the haemolymph. The individuals of *C. destructor* weighed 57.66 ± 7 g and were 10.88 ± 0.56 cm long, while the individuals of *C. quadricarinatus* weighed 54.82 ± 8.04 g and were 11.13 ± 0.91 cm long. The animals were supplied by the yabby aquaculture facility located at ‘Fiumefreddo di Sicilia’ (Catania) (eastern of Sicily). The animals were divided into different rectangular tanks (80 L each), acclimatized for two weeks with continuous aeration (O_2_ > 5.0 mg/L) and a constant temperature (21 ± 1 °C), and fed daily with a commercial diet (5% body weight, Malta Cleyton, SA, Mexico) up to 24 h before the sampling of haemolymph.

### 2.2. Haemolymph Sampling

For haemolymph sampling, each animal was anesthetized in ice for 10 min. All haemolymph samples were taken by inserting a 21-gauge needle into the pericardial sinus at the base of the first abdominal segment.

The haemolymph was obtained from 15 individuals of each species. The samples were taken with anticoagulant by sampling the haemolymph in 0.2 M sodium cacodylate with 1% glutaraldehyde in a 1:1 ratio (1 mL in total). Then, 100 µL of the samples were used for the total haemocytes count and flow cytometer cell sorter. The remaining sample was immediately centrifuged at 800× *g* for 10 min at 4 °C to separate the cells from the supernatant. After centrifugation, a cellular pellet and cell-free samples (CLF) were obtained and used for enzymatic assays. Another 15 individuals for each species were used to evaluate total protein, osmolality and pH values on cell-free samples. In these cases, the haemolymph sampling was carried out without anticoagulant and immediately centrifuged at 800× *g* for 10 min at 4 °C to avoid coagulation.

### 2.3. Total Haemocytes Count and Flow Cytometer Cell Sorter

The total haemocyte count (THC; the number of haemocytes per mm^3^) was determined using a Neubauer haemocytometer chamber. Moreover, the flow cytometer cell sorter (FACS analysis by Epics XL™ flow cytometer with Expo32 software Beckman Coulter, Fullerton, CA, USA) evaluation was performed on each sample to classify cell types and the percentage differences between them. In particular, 500 µL of cells in suspension was used and for each sample, at least 10,000 events were analyzed. Results were expressed using cell cytograms showing the granularity (SS value) and the proportional size (FS value) of cells; moreover, histograms were used to show the percentages of each cell subpopulation. Three cellular subpopulations were defined by their size and complexity and were electronically gated and sorted with FACS.

### 2.4. Enzymatic Assay

The enzymatic activities of the alkaline phosphatase (AKP) and esterase (EST) were performed in the cellular lysate (CL) and cell-free samples of each individual. The cellular pellet of each individual was individually lysed using the minimum RIPA buffer 1X added with antiprotease 1:200 (200 µL). In particular, each sample was pottered for at least 5 min, sonicated for 2 min, and centrifuged at 15,500 rpm for 20 min and 4 °C. The enzymatic activities were evaluated by incubating 50 μL of the sample with 50 μL of buffer in 96-well plates [35]. The buffers for the esterase and phosphatase activity were different. For esterase, the buffer was prepared using 0.4 mM of *p*-nitrophenyl myristate (Sigma-Aldrich, Saint Louis, MO, USA) in a solution of 100 mM ammonium bicarbonate and 0.5% Triton X-100 (pH 7.8); for the alkaline phosphatase activity, the buffer was produced using 4 mM of *p*-nitrophenyl phosphate (Sigma-Aldrich, Saint Louis, MO, USA) in a solution of 100 mM ammonium bicarbonate and 1 mM of MgCl_2_ (pH 7.8). The activity of the two enzymes was measured 3 times every 5 min for 1 h using a spectrophotometer (GloMax^®^-Multi Detection System; Promega Corporation, Madison, WI, USA).

The enzymatic activities were expressed in U/μg of protein and calculated using the formula:

Enzymatic activity = Abs/min × 1000/Eb × Vf/Vi

where Abs/min is the absorbance value obtained for each sample divided by the time at which the measurement was taken (60 min), Vf indicates the final volume of the plate well, Vi represents the initial volume of the plate well, and Eb is an experimental constant (16.4 for the esterase and 18.4 for alkaline phosphatase activities).

### 2.5. Total Protein, Osmolality and pH Evaluation

The total protein was evaluated in cell-free samples of each individual obtained during sampling of haemolymph without anticoagulant (haemolymph serum). In detail, the total protein was evaluated using the Bradford method [36]. Bovine serum albumin (BSA) was used as the protein standard.

Osmolality was estimated with an osmometer (Roebling, Messtechnik, Berlin, Germany). The pH was measured on each cell-free sample using a pH meter with a microelectrode (pH 8 bench meter, XS Instrument).

### 2.6. Statistical Analysis

To determine significant differences between the two *Cherax* species for each haemolymphatic parameter analyzed, an unpaired t-test was used (Statistica 8). Three replicas were performed for each species (five individuals for each replica). Each sample for each replica was analyzed three times.

## 3. Results

**Figure 1:**
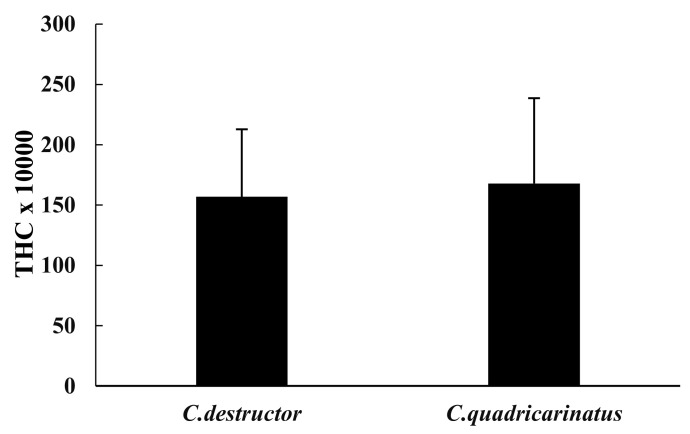
Total haemocyte counts (THC) of *C. destructor* and *C. quadricarinatus* are shown as mean ± SD.

The total haemocyte count (THC) showed very similar values in both species (1.568 × 10^3^ ± 560 × 10^3^ in *C. destructor* and 1.678 × 103 ± 707 × 10^3^ in *C. quadricarinatus*, Figure 1) and were not significant (*p* = 0.70).

**Figure 2:**
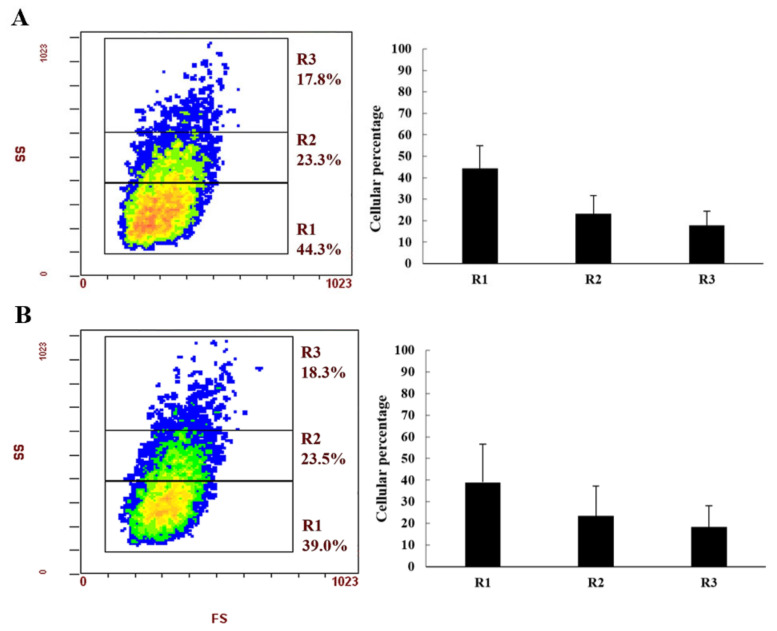
Haemocyte subpopulations of *Cherax destructor* (**A**) and *Cherax quadricarinatus* (**B**) classified by FACS analysis expressed using cell cytograms pointing to the granularity (SS value) and the proportional size (FS value) of cells. On the right, the histogram shows the percentages of each cell subpopulation of the three different regions.

Three subpopulations of haemocytes were classified in both species according to cell complexity (SS) and cell size (FS) (Figure 2A,B). In detail, region 1 (R1) shows cells characterized by low complexity, region 2 (R2) are the more complex cells, and region 3 (R3) are the most complex cells. In both species, the percentage of haemocytes of the three regions is very similar. The highest percentage was observed for cells in R1, followed by R2 and R3 regions, respectively.

**Figure 3:**
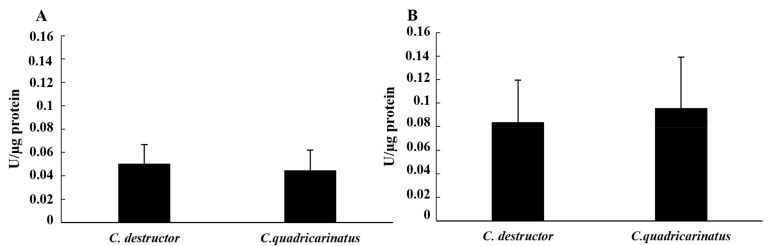
Esterase activity (**A**) and alkaline phosphatase (**B**) activity in the cellular lysate of *C. destructor* and *C. quadricarinatus* are shown as mean ± SD.

The activities of the enzymes of the hydrolase class were evaluated on the cellular lysate and cell-free samples of 15 individuals of both Cherax species after haemolymph collection using anticoagulant. The values obtained from the cellular lysate showed a lower value for the esterase activity compared to the alkaline phosphatase activity in both species. In particular, the esterase activity showed similar values between the two species (0.050 ± 0.016 in *C. destructor* and 0.044 ± 0.017 in *C. quadricarinatus*). On the other hand, the alkaline phosphatase activity was 0.083 ± 0.036 in *C. destructor* and 0.096 ± 0.043 in *C. quadricarinatus* (Figure 3A,B). In both cases, the differences were not significant (*p* = 0.47 for esterase and *p* = 0.51 for alkaline phosphatase).

**Figure 4:**
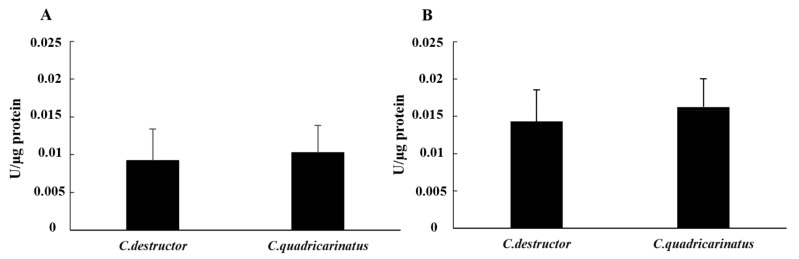
Esterase activity (**A**) and alkaline phosphatase (**B**) activity in cell-free haemolymph of *C. destructor* and *C. quadricarinatus* are shown as mean ± SD.

The hydrolase enzymes in the cell-free samples of both species showed a similar pattern without significant differences (*p* = 0.29 for alkaline phosphatase and *p* = 0.55 for esterase). In fact, the activity of the esterase enzyme in both species was lower than the alkaline phosphatase. Furthermore, in both species, the enzymatic activity in the cell-free haemolymph was lower than those observed in the cellular lysate. In detail, the activity of the esterase enzyme was 0.009 ± 0.004 in *C. destructor* and 0.010 ± 0.004 in *C. quadricarinatus*. Regarding the alkaline phosphatase, in *C. destructor* the activity was 0.014 ± 0.004, while in *C. quadricarinatus* the activity was 0.016 ± 0.004 (Figure 4A,B).

**Figure 5:**
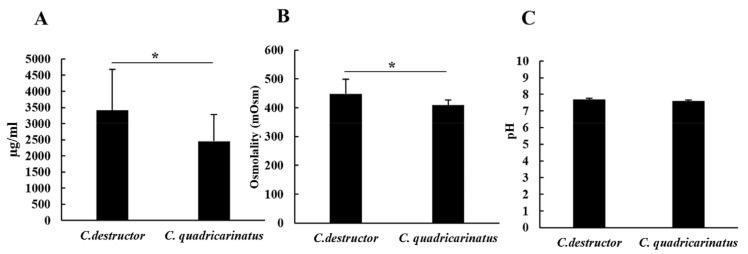
Total protein (**A**), osmolality (**B**), and pH values (**C**) in the haemolymph of *C. destructor* and *C. quadricarinatus* are shown as mean ± SD. (*p < 0.05).

In the haemolymph of the 15 individuals of both species sampled without anticoagulant, total protein, osmolality and pH were also evaluated. The total protein values did not differ much between the two species but were significantly higher (*p* < 0.05) in *C. destructor* (3409 ± 1276 µg/mL) with respect to *C. quadricarinatus* (2455 ± 824 µg/mL) (Figure 5A). In regards to the osmolality, the values were very similar between the two species, even if significantly higher (*p* < 0.05) in *C. destructor*. The osmolality values were 448 ± 51.41 mOsm in *C. destructor* and 409 ± 18.75 mOsm in *C. quadricarinatus* (Figure 5B). In the end, the pH values in the haemolymph were very similar between the two species (7.66 ± 0.095 in *C. destructor* and 7.56 ± 0.105 in *C. quadricarinatus*, Figure 5C).

## 4. Discussion

Crustaceans are very important in aquaculture, but to date, due to the high culture densities, the animal’s diseases are increasing, causing consequential economic damage [37]. As in all invertebrates, including crustaceans, the innate immune system is a very important defense mechanism in which haemocytes play a key function against microorganisms’ infections [38]. Until now, little is known about the health management of the yabby culture, and knowing the values of some haemolymphatic parameters of organisms in good health status could play an important role. One of the most important haemolymphatic parameters useful to understand the health status of organisms is THC, which refers to the number of haemocytes in circulation in the haemolymph of invertebrate species. The immune system is based on circulating haemocytes, which play an important role in immunity surveillance and is used to evaluate the health status of organisms. Several studies in the literature used THC to evaluate the effects of stressful conditions on invertebrates [39,40,41,42,43,44]. Our results showed that THC values were similar between the two species and were in agreement with the values found by [18,45,46]. Using flow cytometer cell sorter (FACS—an extremely important technique that allows thousands of cells to be analyzed in seconds), we classified cell subpopulations of haemocytes from both *Cherax* species. In literature, haemocyte populations in crustaceans were classified, in general, following the morphological characterization according to the size of the cell and the structural complexity of the cytoplasm [47]. Our results are in agreement with reports by [18] and [48], which used microscopic observation and flow cytometry analysis to describe three cellular subpopulations: hyaline, semigranular, and granular. The granulocytes showed higher granularity values than the semigranular and hyaline cells. Moreover, in this study, we found that in both species, the cell population is composed of a higher percentage of hyaline cells, followed by semigranular cells and granular cells, respectively. These data agree with a study by [18] regarding *C. destructor* and were different from what was reported by [48] for *C. quadricarinatus*. In fact, these authors showed that in *C. quadricarinatus* the semigranular and granular cells are the two largest groups of haemocytes compared to the hyalines, which constituted the lower portion, since hyaline cells are rare and immature and are released into the haemolymph under certain conditions, causing a variation of the latter between species and specimens [47]. On the other hand, other authors did not show evidence that the hyaline cells are immature or undifferentiated cells, as in *Penaeus monodon* [49]. The hyaline cells and the granular cells (semigranular and granular) seem to be two morphologically and functionally distinct cell lineages. Moreover, semigranular cells are a transitional form between granular and hyaline cells, which are considered precursors of the first two [50]. Therefore, the higher percentage of hyaline cells found in our study may be due to this. Nonetheless, the flow cytograms obtained in this study were comparable to those observed in other crustacean species, such as *Carcinoscorpius rotundilata* [51] and *Cancer borealis* [47]. 

In addition to cell-mediated responses, defense mechanisms are based on a mediated humoral response involving humoral factors produced and released by immune cells, such as enzymes of the hydrolase class. Regarding enzymatic activity, esterase is one of the most common biomarkers used in aquatic organisms to understand the effects of stressful conditions [52,53,54,55,56,57,58], perform the hydrolysis of the ester bond, and are present in different forms for different substrates [56]. Alkaline phosphatase (AKP), on the other hand, is a metalloenzyme that catalyzes the nonspecific hydrolysis of phosphate monoesters [59]. In stressful conditions, AKP and other enzymes are involved in the degradation of carbohydrates, foreign proteins and lipids to protect the individual also, for example, against pathogen infections [60,61], and could be useful to study the immune status of invertebrates [58,62,63]. In literature, no one analyzed this parameter in the Cherax species, however, other studies evaluated it in other invertebrates’ species. In this study, these enzymatic activities were evaluated in the haemolymph, cellular lysate and in cell-free samples, showing very similar values in both *Cherax* species. In agreement with what was observed in sea cucumbers, these enzymes showed a higher activity level in CL than in CLF [5]. Moreover, the alkaline phosphatase was higher with respect to the esterase enzyme class. The knowledge of the physiological levels of these enzymes is important because they play a fundamental role in the immunity of aquatic organisms. Alkaline phosphatase, for example, has been shown to be involved in many biological processes of aquatic organisms, such as energy metabolism and immune response [64,65,66,67,68]. Enzymes are just some of the proteins present in the haemolymph; in reality, there are other proteins, as indicated by the total protein concentration (TPC) results, each with a different function and physical and biochemical properties that change in physiological and pathological conditions. In addition to performing an enzymatic role, they are involved in coagulation, in the activation of profenoloxidase, and in antimicrobial activity [69,70,71]. In the literature, several authors describe the variation of TPC levels in fish and invertebrates subjected to different stress conditions [32,72,73,74,75]. Our TPC values were significantly higher in *C. destructor* than in *C. quadricarinatus*. However, in both cases, the values obtained were lower than the values reported by other authors in the same species [45,76], likely due to the different methods used to prepare the samples and the assay used to evaluate the TPC. Osmolality is another important biomarker, being that freshwater crustaceans are adapted for active ion uptake and a reduction in passive ion loss to maintain osmotic balance [77], and have been used in ionoregulatory studies, particularly to model ion transport in gills [78]. The patterns of ionoregulation in freshwater crustaceans differ from those in freshwater fish [79], and a change in environmental ion concentrations could be dangerous to the body [80,81] as well as alter the physiological pH of the haemolymph [34]. This issue is important if we consider that in aquatic crustaceans, the acid-base balance between the animal and its environment is mainly regulated by cation/H+ and anionic exchangers [82,83,84]. Regarding osmolality, the values of haemolymph in *C. destructor* were very similar to the values obtained by [21]. Moreover, the osmolality was similar to that obtained in other freshwater crustacean species, like *Procambarus clarkii* [1]. In the end, the pH values of haemolymph obtained in our study agreed with the values shown by [85] in *C. destructor*. The values of osmolarity and pH were comparable with other freshwater species, such as *Macrobrachium rosembergii,* as observed by [86]. Knowing these values is important because, in crustacean species, exposure to stressful conditions (e.g., pollutants) can disrupt the ionic balance and cause extracellular acid-base changes, as observed in *Carcinus maenas* by [87].

## 5. Conclusions

To date, providing reference levels of haemolymphatic biochemical parameters typical of the state of health of aquaculture animals becomes important for the maintenance of farms. The farms of the freshwater *Cherax* species are now widespread, and the knowledge of their health status becomes important at an economical level. To date, no scientific study in the literature has provided information about the levels of some haemolymphatic biochemical parameters (typical of the evaluation of the health status of aquatic invertebrates) of these species. For the first time in this study, we provided THC levels, information on haemocytes subpopulations, levels of esterase and phosphatase enzymes in cells and in cell-free, and levels of protein concentration, osmolarity and pH. We have provided baseline values and demonstrated that there are no significant differences in these parameters between the two most economically important *Cherax* species, except in the case of osmolarity and protein concentration, probably due to a species-specific response. In the future, aquaculture plants will need to evaluate the health status of these animals will be able to evaluate these biochemical haemolymphatic parameters and understand whether the health status will be compromised or not, thus monitoring animal welfare. These parameters will provide information on the correct maintenance of the two freshwater shrimp species.

## Data Availability

The data presented in this study are available on request from the corresponding author (mirella.vazzana@unipa.it). The data are not publicly available due to privacy.
